# Docking and Molecular Dynamic Investigations of Phenylspirodrimanes as Cannabinoid Receptor-2 Agonists

**DOI:** 10.3390/molecules28010044

**Published:** 2022-12-21

**Authors:** Abdelsattar M. Omar, Anfal S. Aljahdali, Martin K. Safo, Gamal A. Mohamed, Sabrin R. M. Ibrahim

**Affiliations:** 1 Department of Pharmaceutical Chemistry, Faculty of Pharmacy, King Abdulaziz University, Jeddah 21589, Saudi Arabia; 2 Department of Pharmaceutical Chemistry, Faculty of Pharmacy, Al-Azhar University, Cairo 11884, Egypt; 3 Center for Artificial Intelligence in Precision Medicines, King Abdulaziz University, Jeddah 21589, Saudi Arabia; 4 Department of Medicinal Chemistry, School of Pharmacy, Virginia Commonwealth University, 800 East Leigh Street, Richmond, VA 23298, USA; 5 Institute for Structural Biology, Drug Discovery and Development, Virginia Commonwealth University, 800 East Leigh Street, Richmond, VA 23298, USA; 6 Department of Natural Products and Alternative Medicine, Faculty of Pharmacy, King Abdulaziz University, Jeddah 21589, Saudi Arabia; 7 Department of Chemistry, Preparatory Year Program, Batterjee Medical College, Jeddah 21442, Saudi Arabia; 8 Department of Pharmacognosy, Faculty of Pharmacy, Assiut University, Assiut 71526, Egypt

**Keywords:** fungi, phenylspirodrimanes, *Stachybotrys*, cannabinoid receptors, industrial development, molecular docking, life on land

## Abstract

Cannabinoid receptor ligands are renowned as being therapeutically crucial for treating diverse health disorders. Phenylspirodrimanes are meroterpenoids with unique and varied structural scaffolds, which are mainly reported from the *Stachybotrys* genus and display an array of bioactivities. In this work, 114 phenylspirodrimanes reported from *Stachybotrys chartarum* were screened for their CB2 agonistic potential using docking and molecular dynamic simulation studies. Compound **56** revealed the highest docking score (−11.222 kcal/mol) compared to **E3R**_6KPF (native agonist, gscore value −12.12 kcal/mol). The molecular docking and molecular simulation results suggest that compound **56** binds to the putative binding site in the CB2 receptor with good affinity involving key interacting amino acid residues similar to that of the native ligands, **E3R**. The molecular interactions displayed π–π stacking with Phe183 and hydrogen bond interactions with Thr114, Leu182, and Ser285. These findings identified the structural features of these metabolites that might lead to the design of selective novel ligands for CB2 receptors. Additionally, phenylspirodrimanes should be further investigated for their potential as a CB2 ligand.

## 1. Introduction

The endocannabinoid system (ECS) is a complex cell-signaling system involved in many functional activities in the body. The ECS consists of cannabinoid receptor 1 (CB1) and cannabinoid receptor 2 (CB2), which are both G-coupled receptors. CB1 is primarily expressed in the central nervous system (CNS). CB2 is expressed in the peripheral immune system. [1,2] ECS has attracted attention as a pharmacological target for several pathological conditions, such as pain, neurodegenerative, and autoimmune disorders [3]. Selective targeting of CB2 is crucial for the development of peripheral system-acting analgesics as it lacks the psychotropic adverse effects typically observed with CB1 agonists. [4] Additionally, the CB2 receptor is a substantial target for discovering therapeutic agents for various disorders such as atherosclerosis, cardiometabolic disorder, osteoporosis, neuro-inflammation, renal ischemia-reperfusion injury, and cardiac ischemia. [5] However, difficulties in achieving target selectivity due to the high sequence identity between CB1 and CB2 have hindered the development of CB2-selective agonists [2,6]. Therefore, the development of novel selective cannabimimetic agents represents a beneficial therapeutic strategy for treating various pathological conditions.

In silico study is a computational tool that enables the assessment of the therapeutic capacity of various metabolites using bioinformatic techniques. This can be utilized to discover new drug candidates and the possible mechanisms of their therapeutic efficacy through predicting drug and protein interactions [7].

Recently, interesting insights have been directed at the naturally biosynthesized chemical scaffolds as leads have been identified in the discovery of new ligands for cannabinoid receptors. Many researchers address ‘Life on Land’ as one of the sustainable development goals by focusing on fungal metabolites, including their regulation, biosynthesis, and bioactivities aiming to find solutions for many health- and environment-related issues. Fungi represent a huge gold mine for structurally diversified and bio-active metabolites that have marked contributions in the fields of pharmaceutics, environmental protection, cosmetics, biotechnology, food, and agriculture [8,9,10,11,12,13,14,15]. Studies about the isolation and bio-evaluation of metabolites from fungal sources are occurring at a faster rate, with hundreds of them reported yearly.

**Figure 1:**
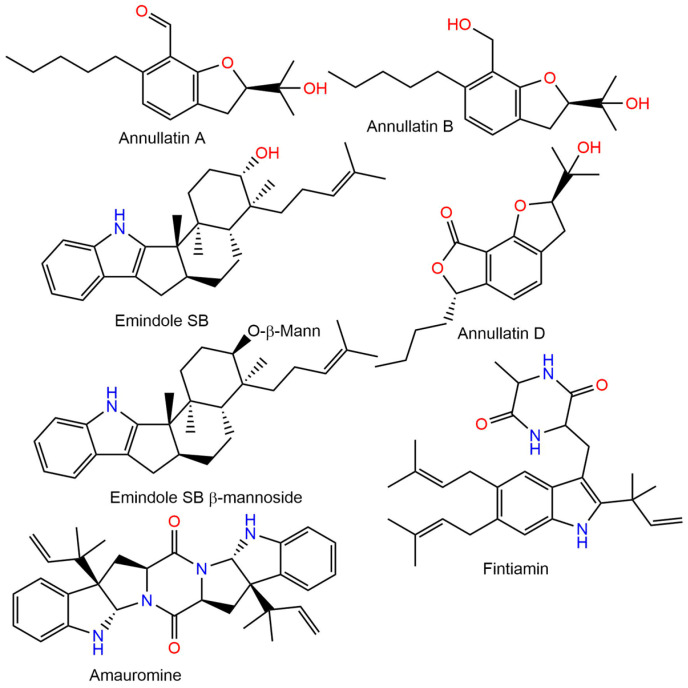
Examples of reported fungal metabolites with cannabinoid receptor modulating potential.

Currently, fungi-derived drug discovery has acquired new focus because of the immense progress that has been observed in genomics, particularly bioinformatics and high-throughput sequencing, in addition to the growing integration of synthetic biology in natural products research [16,17,18,19,20]. Fungi-derived metabolites represent untapped and unexplored domains for cannabinoid receptor-based drug discovery, as well as a pool of leads for the pharmaceutical industry. Therefore, significant interest has been directed to identifying new metabolites from fungi that act on cannabinoid receptors. Several researchers have reported the investigation of the cannabinoid receptors’ modulation capacity of different fungal metabolites (Figure 1).

From the marine-derived *Dichotomomyces cejpii*, emindole SB β-mannoside, and emindole, SB were found to be CB2 antagonist and non-selective CB2/CB1 antagonist, respectively [21]. A study with annullatins A, B, and D from *Cordyceps annullata* revealed the potent agonistic potential of annullatin A on CB2 and CB1, in addition to the CB1 agonistic and CB2 antagonistic effectiveness of annullatins B and D [22]. The radioligand binding investigation showed the high selectivity and affinity of amauromine separated from *Ircinia variabilis*-associated *Auxarthron reticulatum* on the CB1 receptor as a CB1 antagonist [23]. Fintiamin, a lipophilic dipeptide-terpenoid hybrid separated from *Ircinia variabilis-*accompanied *Eurotium* sp, demonstrated marked affinity on the CB_1_ receptor at low micro-molar concentrations [24].

Phenylspirodrimanes are one of the most dominant *Stachybotrys* genus metabolites that possess various structural scaffolds [25]. They are unusual meroterpenoids (terpenyl-phenol) that are distinguished by incorporation through a spiro-furan ring among spirocyclic drimane and phenyl moiety. They are biosynthesized via PKs (polyketide synthases) and terpenoid pathways, as they originate from orsellinic acid and farnesyl diphosphate, respectively. In addition, their dimers are produced from two monomers through C-N or C-C linkage [26]. These metabolites demonstrate various biological potentials, including antiviral, cytotoxic, anti-inflammatory, tyrosine kinase modulatory, and neuroprotective capacities [25]. In our continual goal to explore bioactivities and shed light on these interesting fungal metabolites, 114 phenylspirodrimane derivatives reported from *Stachybotrys chartarum* have been investigated for their effects on ECS. The molecular modeling assessment of these metabolites within the active sites of the CB receptors was performed to study their mode of binding to the target receptor and gain insight into their mechanism of action.

## 2. Results and Discussion

### 2.1. AI (Artificial Intelligence)-Based Target Prediction for Phenylspirodrimanes Derivatives

**Figure 2:**
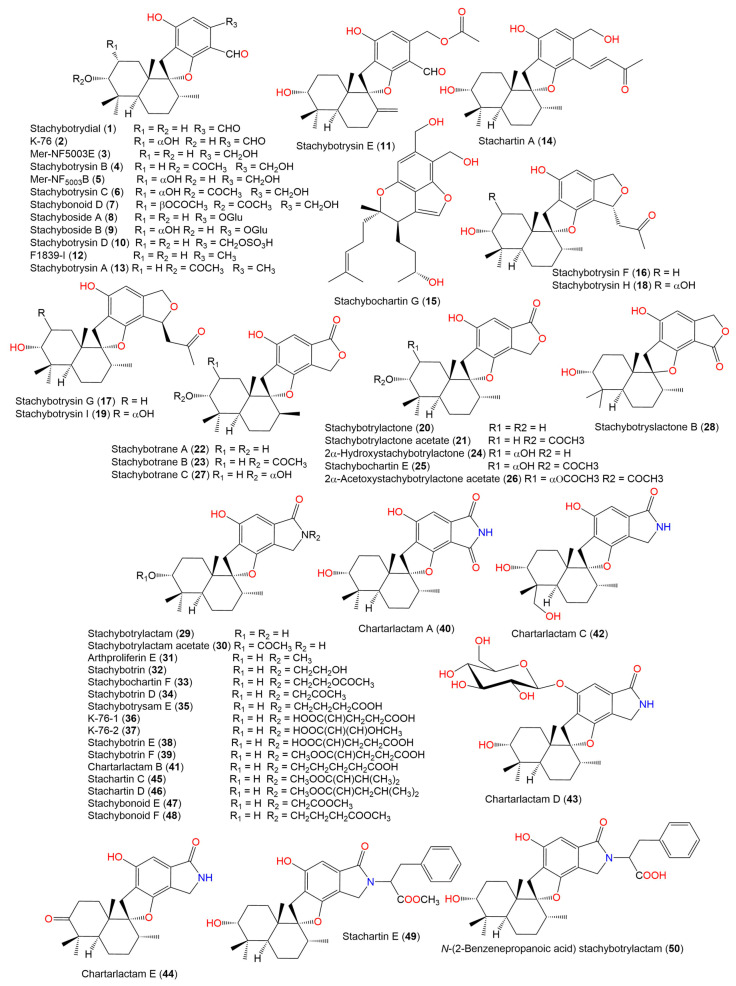
Chemical structures of phenylspirodrimane derivatives (**1**–**50**).

**Figure 3:**
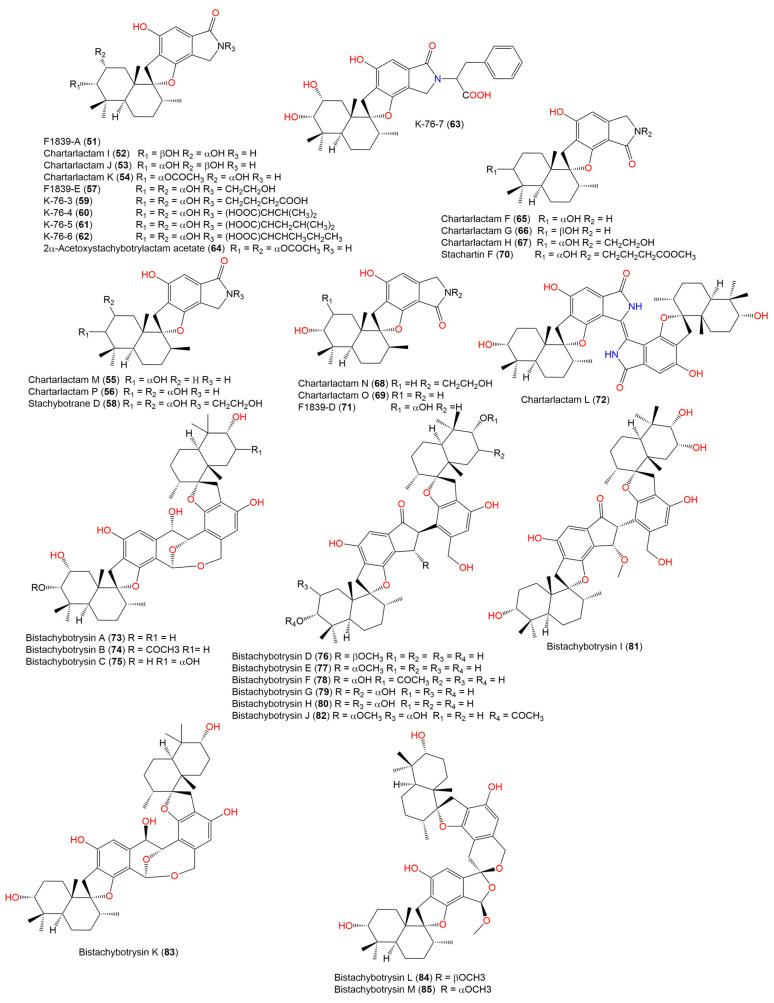
Chemical structures of phenylspirodrimane derivatives (**51**–**85**).

**Figure 4:**
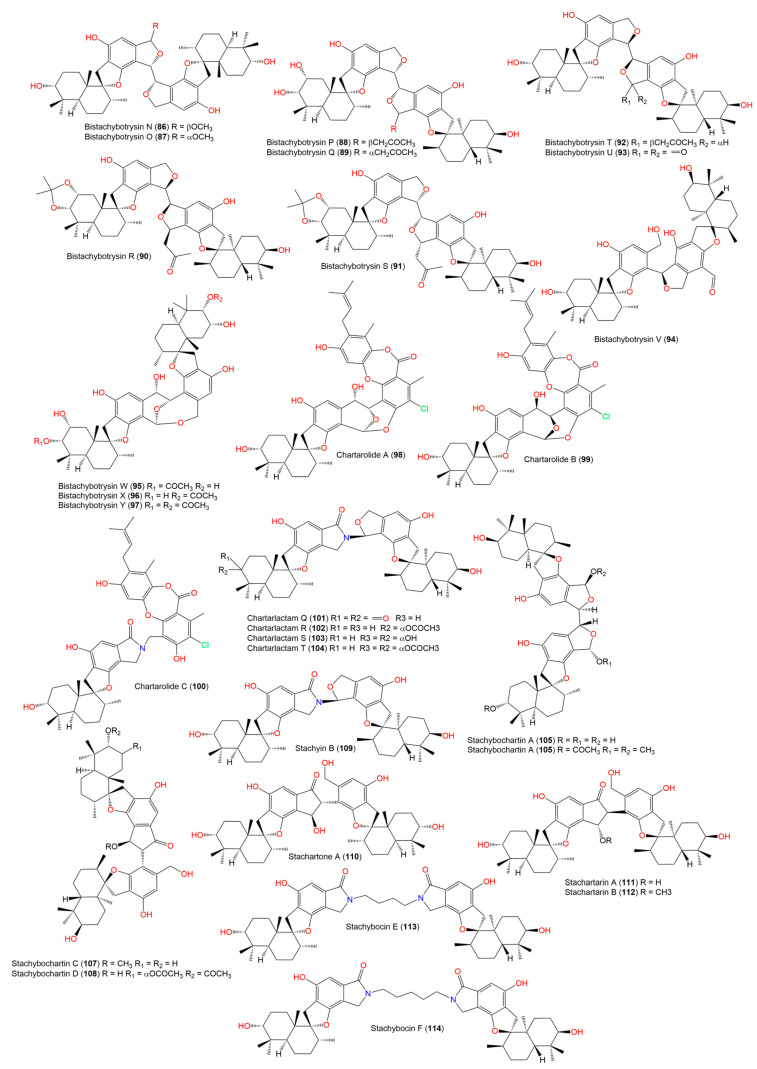
Chemical structures of phenylspirodrimane derivatives (**86**–**114**).

The in silico target prediction web server, SuperPred, was utilized to predict the molecular target for the phenylspirodrimane derivatives (Figure 2, Figure 3 and Figure 4) [27]. SuperPred utilizes the machine learning algorithm for the anatomical therapeutic chemical (ATC) code and target prediction based on a molecular similarity search. [27] In brief, SuperPred suggests the target by translating the query compounds into structural fingerprints and compares it to the compound data set with known bioactivities toward these targets. The drug dataset includes WHO-approved drugs that are classified by a drug classification system, in which each drug’s chemical property is linked to its therapeutic properties and indications, and each classification has an anatomical therapeutic chemical (ATC) code. As a result, if a drug has more than one therapeutic indication, it is assigned an ATC code for each. The WHO has 6300 approved drugs that are linked to over 600,000 targets. SuperPred compares the compound’s fingerprint to that of the WHO-approved drugs based on the notion that compounds with similar physiochemical properties would have similar biological effects. Therefore, if a structural similarity is detected, the ATC code, the possible molecular target(s), and the putative therapeutic indication(s) for that compound are predicted. The target of the most similar compounds in the data set is most likely the target of the query compound. Therefore, for each compound query, a list of targets was predicted. Each target was given a probability and model accuracy score, which represented the likelihood of the compound structure binding with that target. Based on the results, CB2 was selected as the target of choice for the phenylspirodrimane derivatives due to its high probability and model accuracy (Table 1). A crystal structure of the human CB2 receptor in complex with an agonist (PDB ID: **6KPF**) [2] was chosen for the subsequent docking and molecular dynamic (MD) simulation studies.

### 2.2. Molecular Docking Studies

**Figure 5:**
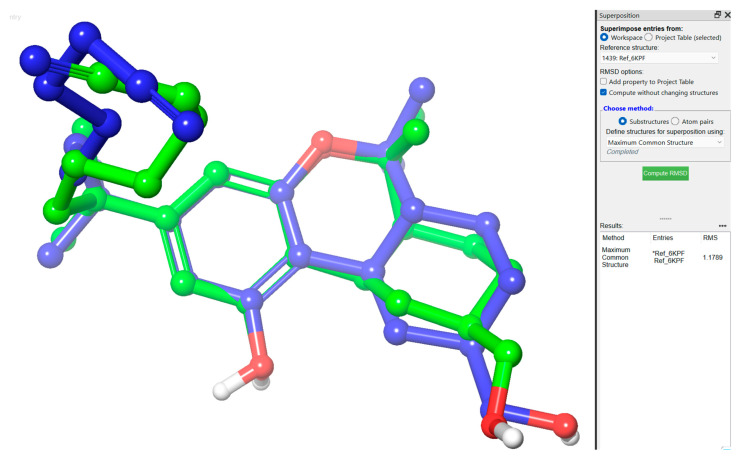
The 3D structure of the redocked E3R superimposed on the co-crystallized E3R.

A total of 114 phenylspirodrimane derivatives in addition to the native agonist, 7-[(6aR,9R,10aR)-1-hydroxy-9-(hydroxymethyl)-6,6-dimethyl-6a,7,8,9,10,10a-hexahydro-6H-benzo[c]chromen-3-yl]- 7-methyloctanenitrile (**AM12033**; PDB ID: **E3R**), bound to human CB2 (PDB ID: **6KPF**) were prepared before the molecular docking experiment. The preparation included converting the 2D structures of the compounds to energy-minimized 3D structures and generating all possible ionization and tautomeric states using Schrodinger’s *LigPrep* tools [28]. The CB2 receptor was retrieved from the protein data bank (PDB) [29], which was then prepared and energy minimized using Schrodinger’s Protein Preparation wizard [28,30]. For the docking experiment, a grid box around the binding site of the co-crystallized native agonist was generated via a *Receptor Grid Generation* tool in Maestro [31,32]. The defined grid box at the binding site located the pocket where the docking was taking place. The docking method was validated by redocking the co-crystalized native agonist and calculating the root-mean-square deviation (RMSD) (Figure 5). The result showed minimal deviation with an RMSD of 1.1789 Å, indicating that the docking method was valid. After that, the docking of the prepared 3D molecular structures of phenylspirodrimane derivatives was carried out using a standard precision scoring function (SP) followed by the extra-precision (XP) scoring function for maximum accuracy [33].

The docking results included a list of compounds (Table 2), which were ranked based on their docking scores and approximated free energy of the binding. Different docking scores were generated for each ligand, including emodel, gscore, and XP gscore. The emodel scoring was used to select the best pose for the docked compounds. The best poses were then ranked based on their gscore, while the XP gscore ranked the poses generated by the Glide XP mode. Generally, Glide ranks the docked compounds using the gscore scoring function. Based on the result listed in Table 2, compound **56** exhibited the highest docking score of −11.222 kcal/mol, which was close to the native agonist gscore value of −12.12 kcal/mol.

**Figure 6:**
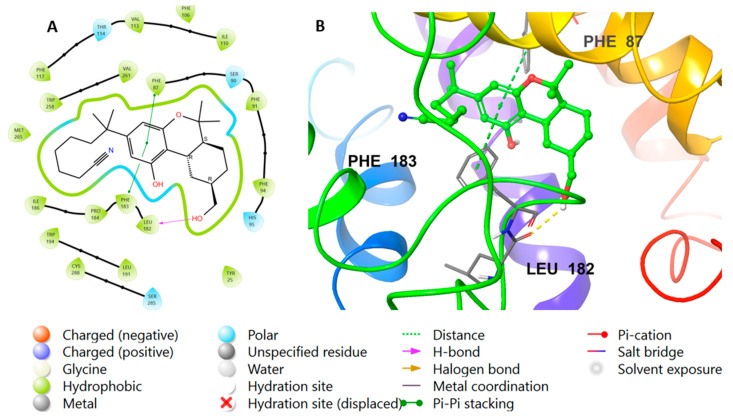
Re-docking of the co-crystallized ligand to validate the docking method. (**A**) The 2D view of the binding interactions of the reference ligand **E3R** complexed with CB2 after re-docking of ligand **E3R** into the CB2 crystal structure. (**B**) The 3D representation for CB2 complexed with ligand **E3R** (green color) after re-docking and interacting with side-chain residues (gray color).

**Figure 7:**
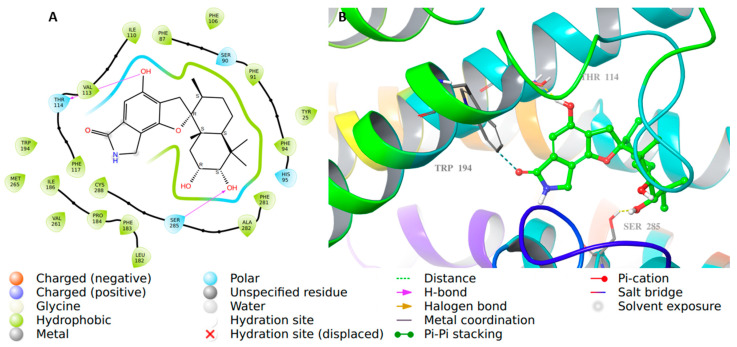
CB2 in complex with **56**. (**A**) The 2D view of the binding interactions of compound **56** complexed with the CB2 crystal structure. (**B**) The 3D representation for CB2 complexed with compound **56** (green color) after re-docking and interacting with side-chain residues (gray color).

The binding interactions were observed with the native agonist E3R (Figure 6) as it interacted through hydrophobic interactions with Phe91, Phe94, and Phe183. In addition, it interacted through hydrogen bond interactions with Ser90, His95, Ser285, and Thr114 and the backbone of Leu182. Similarly, Figure 7 represents the 3D and 2D view of the final preferred docked pose of **56** and the native agonist, respectively. Compound **56** interacted through hydrophobic interactions with Phe91, Phe94, and Phe183. Additionally, it was involved in several hydrogen interactions with His95, Ser285, and Thr114 and the backbone of Leu182 (Figure 7).

### 2.3. Molecular Dynamic (MD) Simulation

**Figure 8:**
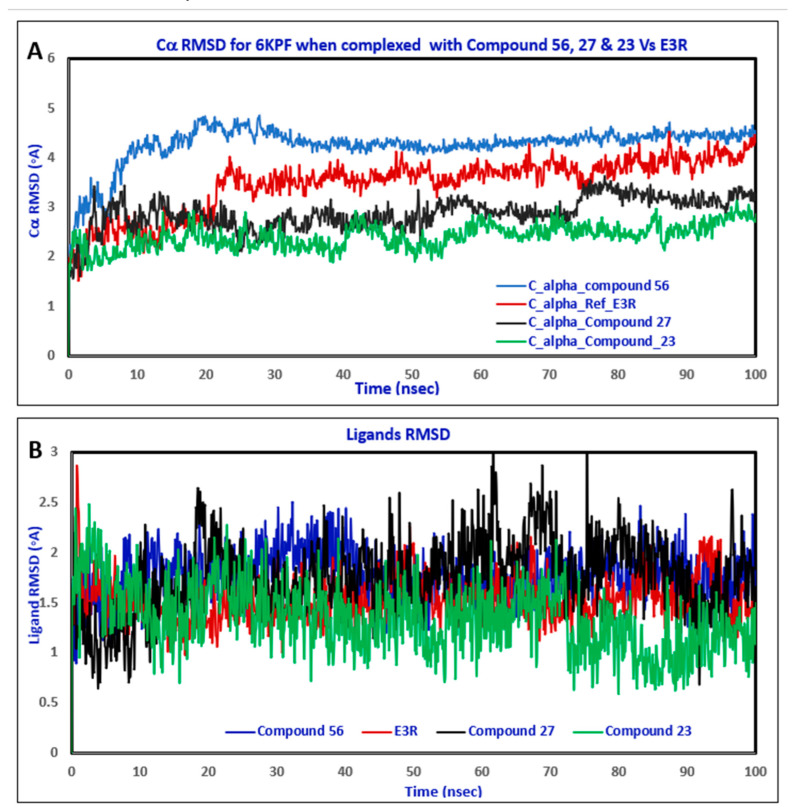
(**A**) The comparison between the RMSD of Cα in the CB2 protein (PDB- ID: 6KPF) plot was obtained when complexed with the reference **E3R** (red color), compound **56** (blue color), compound **27** (black color)_ and compound **23** (green color) during the simulation time (100 ns). (**B**) The comparison of E3R (red color), compound **56** (blue color), compound **27** (black color), and compound **23** (green color) RMSD when complexed with CB2 protein (PDB- ID: 6KPF) during the simulation time (100 ns).

**Figure 9:**
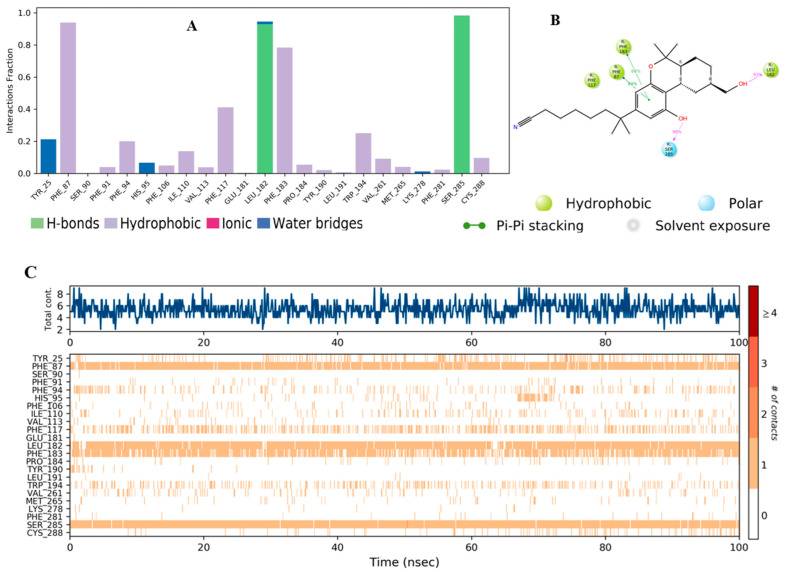
(**A**) CB2 interactions with reference **E3R** throughout the simulation. The interactions between the ligand and protein were classified into ionic, water bonds, hydrophobic, and hydrogen bonds, and each was classified into subtypes. The stacked bar charts were normalized over the course of the trajectory; for example, a value of 0.8 suggested that the specific interaction was maintained during 80% of the simulation time. Values over 1.0 indicated that some protein residue might make multiple interactions of the same subtype with the ligand. (**B**) Schematic diagram exhibiting the detailed 2D atomic interactions of **E3R** with CB2 that occurred > 30% of the simulation time in the selected trajectory (0 through 100 ns). Interactions with >100% occurrence meant that the residues could have multiple interactions of a single type with the same ligand atom. (**C**) A timeline representation of CB2–**E3R** interactions is presented in (**A**). The top panel depicts the total number of specific interactions of the protein with the ligand during its trajectory course. The bottom panel shows the residues’ interactions with the ligand in each trajectory frame. The dark orange color indicates the presence of more than one interaction between some residues and the ligand.

Once the molecular docking was performed, compound **56** and the native agonist were subjected to MD simulation using Desmond software [34,35]. MD simulation simulates the dynamic behavior of the molecular system under computer-generated physiological conditions to assess the protein–ligand complex stability and binding affinity [36]. The protein–ligand complex stability is assessed by the RMSD plot, which measures the deviation of the protein and ligand atoms inside the binding pocket at the end of the simulation period (100 ns) compared to their initial positions before the simulation at 0 ns [37]. The RMSD plot of the native agonist E3R and compound **56** in Figure 8 and Figure 9, respectively, show the RMSD of CB2 on the left *Y*-axis and the ligand RMSD profile aligned on a protein backbone on the right *X*-axis. Compound **56**’s RMSD (Figure 8) showed an observed insignificant fluctuation with the value of 2.5 Å similar to the one observed with the native ligand (2.5 Å) (Figure 8), which is within the acceptable range of 1–3 Å, indicating a stable binding at the binding pocket throughout the simulation period (Supplementary Materials).

**Figure 10:**
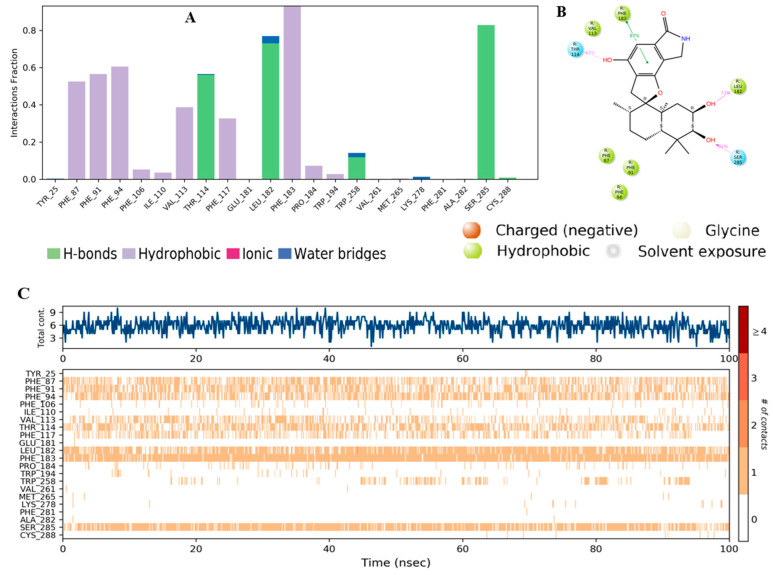
(**A**) TK interactions with compound **56** throughout the simulation. The interactions between the ligand and protein were classified into ionic, water bonds, hydrophobic, and hydrogen bonds, and each was classified into subtypes. The stacked bar charts were normalized over the course of the trajectory; for example, a value of 0.8 suggested that the specific interaction was maintained during 80% of the simulation time. Values over 1.0 indicated that some protein residue might make multiple interactions of the same subtype with the ligand. (**B**) Schematic diagram showing the detailed 2D atomic interactions of **56** with CB2 that occurred for > 30% of the simulation time in the selected trajectory (0 through 100 ns). Interactions with >100% occurrence meant that residues might have multiple interactions of a single type with the same ligand atom. (**C**) A timeline representation of CB2–**E3R** interactions is presented in (**A**). The top panel depicts the total number of specific interactions of the protein with the ligand during its trajectory course. The bottom panel showed the residue interactions with the ligand in each trajectory frame. The dark orange color indicates the presence of more than one interaction between some residues and the ligand.

The residue contacts of the native agonist E3R with CB2 (Figure 9A) demonstrated hydrophobic interactions with Phe87 and Phe183 that were maintained for over 90% and 80%, respectively. Other key interactions, including hydrogen bond interactions with Ser285 and Leu182, were noticed, which lasted for over 90% of the simulation time. The detailed interaction with the protein residues (Figure 9B) revealed π–π stacking interactions with Phe87 (84%) and Phe183 (68%). Hydrogen bond interactions with Leu182 (93%) and Ser285 (98%) were also observed. The top panel of Figure 10C demonstrates the total specific interactions between the native ligand and the protein. The bottom panel shows the protein residues that interacted with the ligand at each time point. The dark orange color in the bottom panel was observed with several residues throughout the trajectory, including Phe87, Leu182, Phe183, and Ser 285.

The residue contacts of the CB2 receptor with **56** (Figure 10A) is presented in the form of stacked bar charts that are color-coded based on the interaction types, including hydrogen bonds, hydrophobic, ionic, and water bridges. The stacked bar chart was normalized over the course of a 100 ns trajectory; a value of 0.8 suggested that the specific interaction was maintained during 80% of the simulation time, while values of over 1.0 indicated that the specific interactions were maintained throughout the simulation time with the possibility of some residues having multiple contacts of the same subtype with the ligand. Phe183 had a π–π stacking interaction with compound **56** that occurred for over 80% of the simulation time (Figure 10A,B). Additionally, other hydrophobic interactions were observed with Phe87, Phe91, and Phe94 that were maintained between 50 and 60% of the simulation period. Furthermore, hydrogen bond interactions with Thr114, Leu182, and Ser285 were also noticed and lasted for 60–80% of the simulation time. The 2D schematic representation of **56** interactions with the protein residue was only considered. The interactions occurred for over 30% of the simulation time. The analysis of the binding interactions showed that compound **56** interacted through π–π stacking with Phe183 (87%) and hydrogen bond interactions with Thr114 (56%), Leu182 (73%), and Ser285 (81%). Figure 10C (top plot) displays the total specific interactions between the ligand and the protein, whereas the bottom panel demonstrates the protein residues that interacted with the ligand at each time point. As mentioned earlier, Leu182, Phe183, and Ser285 made over 70% of the interactions with **56**, which is indicated by the dark orange color in the plot throughout the trajectory (Figure 10C).

The comparative analysis of binding interactions with compounds **56** and the native ligand showed that **56** had more total contacts (Figure 10C) than the native agonist (Figure 10C) and this might be due to the structural differences and 3D conformation of the compound **56** inside the binding pocket.

### 2.4. In Silico ADME Properties of Selected Ligand

Compounds with the highest docking score and lowest free binding energy were further analyzed for their drug-likeness and ADME (absorption, distribution, metabolism, and excretion) properties via Maestro’s QikProp Schrodinger module [38]. This module provides the quick and reliable prediction of many physiochemical properties along with other descriptors, such as the number of possible metabolites and the number of reactive functional groups, to evaluate the usefulness of the investigated compounds by describing and determining their drug-likeness, physiochemical properties, and expected toxicity profiles. Additionally, QikProp provides a range for comparing each property to 95% of the known drugs’ properties. The ADMET prediction aids in filtering out the compounds that might pose a problem during the clinical stage of drug discovery and development. As a result, it minimizes the failure in the drug discovery process. The predicted ADMET properties and descriptors for the compounds are presented in Table 3. The results show that all the compounds’ descriptors were within the recommended range.

## 3. Materials and Methods

### 3.1. Target Prediction

The Superped web server was used to determine the molecular targets for the phenylspirodrimane derivatives [27]. SuperPred is a knowledge-based tool that employs machine learning models that use logistic regression and Morgan fingerprints of length 2048 for the ATC code and target prediction of query compounds. Following the target’s prediction, a probability score and a model accuracy score are reported for each target. The probability represents the likelihood that the query compound will bind to a specific predicted target. On the other hand, the model accurately represents the performance accuracy of the used machine-learning model when predicting the specific target for the compound since the model performance differs between targets [27,39].

### 3.2. Ligand and Protein Preparation

Phenylspirodrimane derivatives were prepared for docking studies using Schrödinger’s LigPrep tool [28]. The 2D structures of all the compounds were converted to energy-minimized 3D structures using the OPLS3 force field. Hydrogens were added, and all possible ionization states and tautomeric forms were created at a pH of 7.0 ± 0.2 by Epik; a desalt option was also chosen. H–bonds were optimized by predicting the pKa of ionizable groups using PROPKA. In addition, the X-ray crystal structure of the CB2 receptor (PDB: **6KPF**) was retrieved from the protein data bank and prepared using the protein preparation Wizard in Maestro Schrödinger. The missing hydrogens were added to the residues, the metal ionization state was corrected, and the water molecules >5 Å from protein residues were deleted. Only the subunit bound to the ligand was kept from the multi-subunit proteins. Then, the protein was refined by predicting the pKa of the ionizable residues using PROPKA, and water molecules >3 Å (not involved in the water bridge) were removed. Finally, the restrained minimization of the protein was applied using the OPLS4 force field.

### 3.3. Grid Generation and Molecular Docking

A grid box was defined around the co-crystallized ligand binding site and using Glide’s Receptor-Grid-Generation tool [31]. The phenylspirodrimanes’ docking was carried out inside the assigned grid box using the Ligand Docking tool in the Schrödinger suite [28,32]. The non-polar atoms were set for the VdW radii scaling factor by 1.0, and the partial charge cut-off was 0.25. All docking settings were set to default except for the docking protocol that was first selected as a standard precision (SP) before it was then changed to an extra precession (XP) mode with flexible ligand sampling. The re-docking of the co-crystallized ligand was performed to validate the docking method alongside the investigated phenylspirodrimanes.

### 3.4. MD Simulation

MD simulations were performed using Desmond in the Schrödinger package [34,35]. First, the protein–ligand complexes were retrieved from the docking results. The selected ligand–protein complexes were tuned through the “System-Builder‘‘ tool to generate the solvated system for simulation. The solvent model was set as TIP3P, the selected box shape was orthorhombic, and the box dimensions were 10 Å. In addition, Na ions were added to neutralize the system. The simulation parameters were set up in the Molecular Dynamic tool, where the protein–ligand complexes were evaluated at pH 7.0 ± 0.2 over the 100 ns simulation time, and the ensemble class was set as NPT to maintain the temperature and pressure constant during the run at 300 K and 1.01325 bar, respectively. Simulations were run with the OPLS4 force field. After running the MD simulation, the generated results were analyzed.

### 3.5. ADMET Properties Prediction

The selected compounds were subjected to ADMET prediction using the QikProp- module of the Schrodinger suite [38]. For each compound, a list of descriptors was predicted, including molecular weight (mol_MW), drug-likeness (#Stars), dipole moment (dipole), total solvent accessible surface area (SASA), number of hydrogen bond donors and acceptors (donorHB and acceptHB), predicted octanol-water partitioning (QPlogPo/w), predicted aqueous solubility (QPlogS), estimated binding to human serum albumin (QPlogKhsa), number of the possible metabolites (# metab), predicted blood-brain partitioning (QPlogBB), percentage of human oral absorption, predicted IC50 for inhibiting HERG-K^+^ channels (QPogHERG), central nervous system activity (CNS), and the number of reactive functional groups present (#rtvFG). The predicted values were then compared to the recommended range derived from values determined/observed for 95% of the known drugs.

## 4. Conclusions

Cannabinoid receptors are G-protein-coupled receptors that comprise CB1 and CB2 receptors. CB receptors are implicated in many physiological and pathophysiological processes in the body. Several drug discovery efforts have been directed towards selectively targeting CB2 receptors with agonists, primarily due to their promising therapeutic potential for treating pain and inflammation without the psychological side effects that are present with the targeting CB1 receptor. In this paper, the in silico approach, including molecular docking and MD simulation combined with the ADME prediction study, was utilized to explore the binding interaction and affinity of phenylspirodrimanes compounds to the CB2 receptor. The results showed compound **56** to be a potential candidate as it was shown to have a high affinity to the receptor with binding interactions similar to that of the native agonist. Additionally, compound **56** was found to be stable at the binding site in the simulated aqueous physiological environment. The obtained results could have a great contribution to the development, design, and discovery of potent and selective CB2 ligands. However, further in vitro, in vivo, and mechanistical investigations are warranted.

## Figures and Tables

**Table 1:** Prediction of target probability and model accuracy for phenylspirodrimane derivatives against CB2 using SuperPred target prediction web server.

Compound	Probability *	Model Accuracy **
27	58%	97%
56	68%	97%
79	84%	97%
80	87%	97%
81	84%	97%
82	87%	97%
87	83%	97%
89	85%	97%
96	81%	97%
102	80%	97%
107	85%	97%
108	79%	97%
109	87%	97%
110	85%	97%
111	85%	97%
112	85%	97%
113	91%	97%
114	83%	97%

* The probability of the test compound binding to a specific target, as determined by the respective target machine learning model. ** The accuracy of the performance of the prediction model displaying the 10-fold cross-validation score of the respective logistic regression model, as the model performance varied between different targets.

**Table 2:** Docking results of phenylspirodrimane derivatives with human CB2 receptor (PDB: 6KPF).

Compound	Docking Score	Glide Gscore	Glide Emodel	XP GScore
E3R_6KPF	−12.12	−12.12	−64.537	−12.12
56	−11.22	−11.222	31.932	−11.222
27	−11.106	−11.108	25.961	−11.108
23	−10.506	−10.507	37.205	−10.507
15	−10.193	−10.193	−1.513	−10.193
55	−10.17	−10.171	36.385	−10.171
22	−9.957	−9.958	25.109	−9.958

**Table 3:** Selected compounds were analyzed for their ADME (absorption, distribution, metabolism, and excretion) properties using via QikProp.

Molecule	Recommended Range	Stachybochartin G (15)	Stachybotrane A (22)	Stachybotrane B (23)	Stachybotrane C (27)	Chartarlactam M (55)	Chartarlactam P (56)	Reference
#stars	0–5	0	0	0	0	0	0	0
#rtvFG	0–2	0	0	1	0	0	0	0
CNS	−2 (inactive) +2 (active)	−2	−1	−1	−2	−2	−2	−1
mol_MW	130.0–725.0	388.503	386.487	428.524	402.486	385.502	401.502	421.581
SASA	300.0–1000.0	677.156	595.43	641.055	605.78	613.265	622.968	738.361
volume	500.0–2000.0	1269.411	1144.614	1260.485	1165.663	1166.122	1185.684	1374.214
donorHB	0.0–6.0	3	2	1	3	3	4	2
accptHB	2.0–20.0	6.35	6.2	6.5	7.9	5.7	7.4	5.2
QPlogPw	4.0–45.0	11.089	11.101	10.117	14.344	12.255	15.497	9.386
QPlogPo/w	−2.0–6.5	3.415	2.834	3.449	1.887	2.831	1.866	5.171
QPlogS	−6.5–0.5	−4.352	−4.597	−5.248	−4.125	−4.933	−4.428	−6.376
QPlogHERG	concern below −5	−4.551	−3.577	−3.704	−3.65	−3.817	−3.882	−5.34
QPPCaco	<25 poor, >500 great	442.716	393.449	296.585	150.047	247.784	94.544	1603.15
QPlogBB	−3.0–1.2	−1.453	−0.804	−0.939	−1.254	−1.032	−1.495	−0.767
#metab	1–8	8	4	3	5	4	5	2
QPlogKhsa	−1.5–1.5	0.287	0.467	0.709	0.187	0.537	0.244	0.939
Human Oral Absorption	1, 2, or 3 for low, medium, or high	3	3	3	3	3	3	1
Percent Human Oral Absorption	>80% is high <25% is poor	94.3	89.983	91.385	76.944	86.374	73.229	100

## Data Availability

Not applicable.

## Supplementary Materials

The following supporting information can be downloaded at: https://www.mdpi.com/article/10.3390/molecules28010044/s1, Details of molecular dynamics simulation for **E3R**, compound **56**, compound **27**, and compound **23**.
